# New functionally dioecious bush tomato from northwestern Australia, *Solanum ossicruentum*, may utilize “trample burr” dispersal

**DOI:** 10.3897/phytokeys.63.7743

**Published:** 2016-05-03

**Authors:** Christopher T. Martine, Jason T. Cantley, Emma S. Frawley, Alice R. Butler, Ingrid E. Jordon-Thaden

**Affiliations:** 1 Department of Biology, Bucknell University, 1 Dent Drive, Lewisburg, PA, USA; 2 University and Jepson Herbaria, University of California, Berkeley, CA, USA

**Keywords:** Bush tomato, cryptic dioecy, inaperturate pollen, Keep River National Park, Kimberley, Mirima National Park, new species, Northern Territory, Solanum, Solanum dioicum, Solanum sp. Tanami, undergraduate research, Western Australia

## Abstract

A new Australian species of functionally dioecious bush tomato of Solanum subgenus Leptostemonum is described. *Solanum ossicruentum* Martine & J.Cantley, **sp. nov.**, is thought to be allied with members of the problematic “Dioicum Complex” lineage, but differs in its short silvery indumentum, long calyx lobes, larger stature, and an unusual fruit morphology that may represent “trample burr” seed dispersal. The species occurs in a range extending from the eastern Kimberley in Western Australia to far northwestern Northern Territory and has been recognized for decades as a variant of *Solanum dioicum* W.Fitzg. Specimens of this species were previously referred to by D.E. Symon and others as *Solanum dioicum* ‘Tanami.’ Ex situ crossing studies and SEM images of inaperturate pollen grains produced in morphologically hermaphrodite flowers indicate that this taxon is functionally dioecious. The scientific name was chosen with the help of 150 seventh grade life science students from Pennsylvania, USA.

## Introduction

Dioecy in *Solanum* (Solanaceae) is one of the more fascinating phenomena in plant reproductive biology (Knapp et al. 1998). Species exhibiting this breeding system do so in a functional sense whereby male plants bear morphologically staminate flowers and female plants bear morphologically hermaphrodite flowers with anthers that (typically) produce inaperturate pollen. First described using crossing studies and SEM imaging for the Mesoamerican *Solanum appendiculatum* (Anderson 1979, Anderson and Levine 1982, Levine and Anderson 1986, Zavada and Anderson 1997), functional dioecy has now been identified in around 20 *Solanum* taxa (Barrett 2013, Martine et al. 2013). The highest incidence of functional (also referred to as “cryptic”) dioecy in *Solanum* occurs in Australia, where Anderson and Symon (1989) unequivocally confirmed the condition in nine species (based on Symon 1981) via ex situ crossing experiments. Since that time, several new and putative dioecious *Solanum* species have been recognized in Australia, nearly all of them members of the “Dioicum Complex” (Symon 1981, Martine et al. 2006, 2009) in the Kimberley region of Western Australia (Brennan et al. 2006, Barrett 2013, M. Barrett pers. comm.).

*Solanum ossicruentum* Martine & J.Cantley, sp. nov. is one of the many recognizable variants currently included under the broad taxonomic umbrella (Symon 1981; Purdie et al. 1982) of *Solanum dioicum* W.Fitzg.. Identified by collectors (including D.E. Symon and P.K. Latz) since the 1970s as *Solanum dioicum* ‘Tanami’ or *Solanum* sp. ‘Tanami’, this taxon is not only morphologically distinct (Symon 1981, Wheeler et al. 1992), but largely occurs outside of the range of its allied species, extending into the northern edges of the Tanami Desert. Symon (1981) identified three widespread and recognizably different forms of the broadly circumscribed *Solanum dioicum*, identifying ‘Tanami’ as an inland form occupying the “eastern margin” of the species range and noted its distinctiveness in being “closely and densely silvery-pubescent, compact, and extremely prickly.” Here we describe this form as a new species of *Solanum*.

## Methods

Recent observations of the taxon by CTM in Mirima National Park (WA), the Carr Boyd Ranges (WA), and Keep River National Park (NT) are combined here with inferences from plants grown in cultivation from wild-collected seed and herbarium sheets held at the Northern Territory Herbarium, Palmerston (DNA). Seeds were germinated following a 24-hour soak in 1000-ppm gibberellic acid and sown in a controlled growth chamber environment as per Martine et al. (2016). To generate pollen images, fresh pollen mounts from male and female flowers were sputter coated with gold on a Denton Vacuum Desk IV Sputterer (Moorestown, NJ, USA) and examined under a scanning electron microscope (FEI Quanta 400, Hillsborough, OR, USA). Trichome densities were counted under a dissecting scope using 0.25 cm radius holes punched from fresh leaves of seven individual plants (5 leaves per plant and 2 samples per leaf).

## Taxonomic treatment

### Solanum ossicruentum

Taxon classificationPlantaeSolanalesSolanaceae

Martine & J.Cantley sp. nov.

urn:lsid:ipni.org:names:77154529-1

Figs 1 , 2

#### Diagnosis.

This species is distinguished from other dioecious solanums in northwestern Australia by its short silvery indumentum, long calyx lobes, larger and compact stature, and a bony hard mature fruit that remains enclosed in a heavily armed calyx.

#### Type.

AUSTRALIA. Western Australia: Mirima (Hidden Valley) National Park, below upper lookout on Derdbe-Gerring Banan Lookout Trail, 15°45.827'S, 128°45.105'E, 18 May 2014 (staminate and “female” flowers; fruit), *Christopher T. Martine and Rachel F. Martine 4011* (holotype: DNA; isotypes: PERTH, BUPL, CONN)

#### Description.

Clonal, upright woody shrub to 1–2 m tall and 1–2.5 m wide. Single woody stems ca. 2.5 cm diameter from woody rootstock, splitting at about 1/3 of total height to form a Y-shaped or inverted tripod-like growth form, ultimately branching 4–10 times. Overall plant aspect silvery to bluish-green to gray-green, the young growth tomentose-lanate, with older stems woody and gray. Internodes 4.5–8 cm. Stems with short, dense indumentum of stellate trichomes. Prickles straight, long, thin, somewhat sharp, 6–8 mm long, slightly widened at base, abundant and dense (7–15 per cm of internode) on all stems including older woody growth. Leaves 13–23 cm × 4–5 cm, alternate, lanceolate, unarmed; margins entire to undulate; base truncate to rounded, asymmetrical; petiole 10–19 mm long, with scattered prickles; blade soft silvery-blue/gray-green to sage green, concolorous, both sides densely silvery-tomentose (380–560 trichomes per 0.25 cm radius leaf disk); trichomes mostly short stalked, porrect-stellate with short central ray (midpoint). Inflorescences borne on new growth.

*Male* inflorescence a cyme about ca. 4–5 cm long with 2–12 flowers, unbranched, typically with only 2–3 flowers open at a time; peduncle ca. 2–2.5 cm long; rachis 2–2.5 cm long; pedicels ca. 2 mm, unarmed; calyx 5-lobed with or without a few prickles towards the base, the lobes 1.2–1.5 cm long with linear acumens; corolla 3.5–3.8 cm diameter, dark violet, rotate-stellate to rotate, glabrous adaxially and abaxially except for pubescence of minute simple hairs along folds; acumens 0.75–1.25 mm; stamens 5, ca. 9 mm long, equal; anthers ca. 5 mm long, oblong-lanceolate to somewhat tapered, connivent, yellow, poricidal; filaments ca. 4 mm, connate at base; ovary, style, and stigma vestigial, non-functional, and not exserted beyond the stamens.

**Figure 1.**
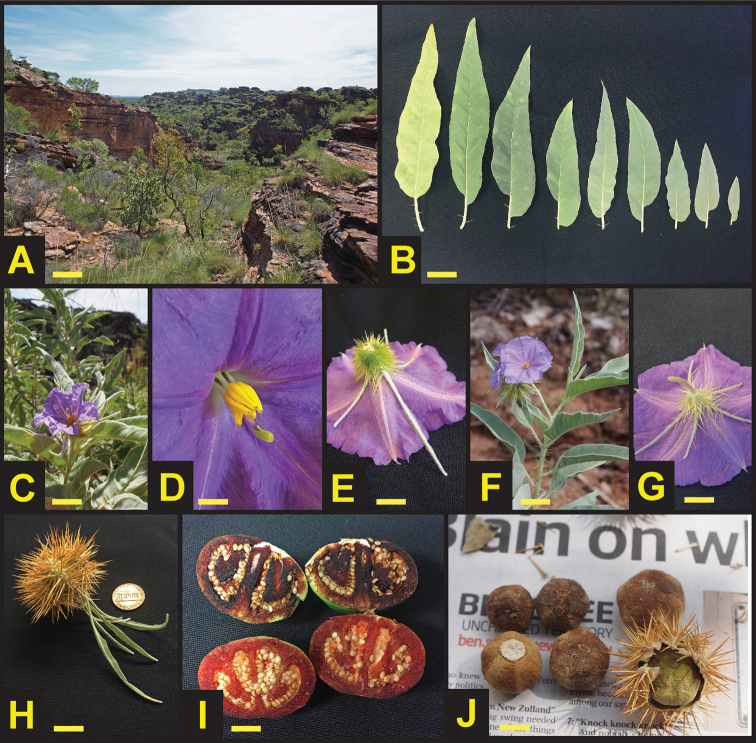
*Solanum ossicruentum* sp. nov. **A** Typical habitat, Mirima National Park, WA **B** Leaf morphology **C** Female individual, Mirima NP **D** Close-up of functionally female (morphologically hermaphrodite) flower **E** Abaxial side of functionally female flower showing elongated calyx lobes **F** Male individual, Mirima NP **G** Male flower, abaxial view **H** Developing fruit within calyx **I** Immature fruits showing blood-red staining at 2 minutes (lower) and 5 minutes (above) after cutting **J** Mature bony fruits removed from calyces and (lower right) as collected from ground beneath plant. Yellow scale bars as follows: 3 cm (**B, C, F**); 1 cm (**D**); 2 cm (**E, G, H, J**); 0.75 cm (**I**). Photos **A**, **C**, **F**, and **J** by C.T. Martine; all others by J.T. Cantley.

**Figure 2.**
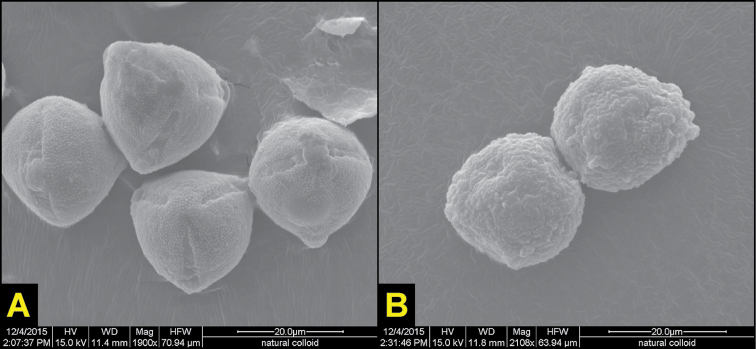
SEM images of *Solanum ossicruentum* sp. nov. pollen grains. **A** Functional pollen produced by male flowers, and **B** Inaperturate pollen produced by morphologically hermaphrodite, yet functionally female, flowers. Images by A. Butler.

Morphologically *hermaphrodite* flowers solitary, functionally *female*, with anthers producing inaperturate pollen (Fig. 2). Female flower on pedicel 0.25–1.0 cm long, armed with small prickles to 2 mm long; calyx densely armed along ribs of tube with long (9–10 mm), straight prickles and stellate trichomes; lobes 1.5–6 cm, narrowly linear, prickly; corolla ca. 3–7 cm diameter, rotate-stellate to stellate-campanulate/funnelform, vibrantly violet, glabrous adaxially and abaxially except for pubescence of minute simple hairs along folds; acumens ca. 1.5–2.0 mm; stamens of same proportions as in male flowers; ovary ca. 4 mm diameter at anthesis, with scattered short, green trichomes; style erect, ca. 14 mm long (including stigmatic surfaces); stigma green, ca. 4 mm long, with slight bifurcation along final 0.5–1.0 mm.

Fruit a berry 1.5–2.5 cm diameter, globose; immature fruit light green, fleshy, with slightly sticky flesh oxidizing from whitish-green to deep blood-red when cut; mature fruit drying to dark green, then chestnut brown, becoming leathery-reticulate in texture and bony hard, weakly six-angled, and loosely retained and partly-enclosed (±75%-enclosed) in calyx, with a 6–8 mm diameter light-colored disk-shaped abscission scar. Fruiting calyx lobes 4.5–7.25 cm long and long-acuminate (acumens breaking off with age), densely armed with sharp prickles 7–8 mm long, tapering to long fine tip, 4–5 prickles per jagged line along ribs and spreading, short stellate-pubescent, more so on calyx ribs and around bases of prickles. Calyx slightly sticky-adherent to fruit when immature, readily separating from fruit as the berry matures, hardens, and shrinks from drying. Fruit and intact calyx ultimately detaching from plant as one light brown, sharply spiny, 3.5–4.5 cm diameter dispersal unit. Seeds ca. 1.5 mm diameter, tan to brown, conspicuously and minutely reticulate, up to 500–650 per fruit.

#### Distribution and ecology.

**Figure 3.**
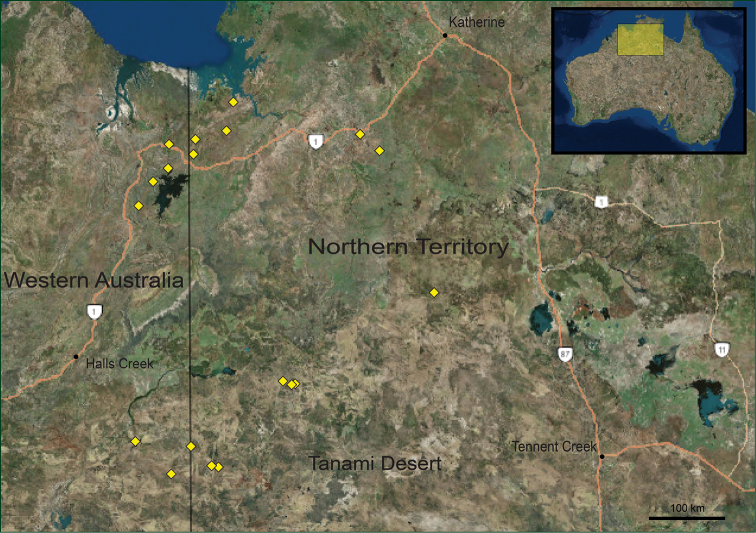
Map showing distribution of *Solanum ossicruentum* sp. nov. accessions held at the Northern Territory Herbarium, Palmerston (DNA) and examined for this description. Map base layer generated from ArcGIS.

*Solanum ossicruentum* is presently known from a wide range of localities in the sub-arid tropical zone of the Northern Territory and eastern Kimberley in Western Australia, including the northern edge of the Tanami Desert (Fig. 3), mostly within the Victoria Bonaparte Terrestrial Bioregion (Australian Government 2012). The species associates closely with red sandstone, quartzite sandstone, and conglomerates (as per Tyler 1996), where it is found on hills, ridges, outcrops, and plateaus, growing in gravel or from fissures in pavement and dissected rock. It has also been collected frequently in steep gorges and washes, as well as at the base of rock formations in sandy levees and alluvial deposits. Among the associated taxa noted on herbarium labels are species of *Triodia* (Poaceae), *Acacia* (Fabaceae), Eucalyptus (Myrtaceae), and *Grevillea* (Proteaceae). Although little is known about its relation with fire (one fruiting collection by Latz is from a recently burned habitat), the species is likely fire tolerant to some degree. Pollination biology of the species is unknown, but, like other Australian congeners, the flowers are likely buzz pollinated by bees in the genera *Xylocopa* and *Amegilla* (see Anderson and Symon 1988, Switzer et al. 2015). A small set (n=8) of ex situ hand pollinations conducted for this study showed that inaperturate pollen produced by functional females does not lead to fruit set when used to pollinate other females – suggesting that, like other dioecious solanums, reproduction in this species is dependent on intersexual outcrossing via biotic pollination. SEM images of the pollen (Fig. 2) confirm that morphologically hermaphrodite flowers produce inaperturate grains incapable of germination.

Seed dispersal appears to follow the relatively uncommon “trample burr” pattern for *Solanum* described by Symon (1979), whereby lightweight fruits enclosed in spiny calyces are carried in the fur of mammals. The fruits of *Solanum ossicruentum* detach enclosed within a long-spiny calyx at maturity, the diaspores gathering in piles on the ground or getting caught in tufts of hummock-forming spinifex grass (*Triodia* spp.) growing below parent plants. In the course of this study, only seeds from mature, bony fruits – the condition they are in when dropped from the plant - proved to be germinable.

#### Uses.

Doonday et al. (2013) describe the use of *Solanum dioicum* (sensu lato), or “salty bush tomato,” by the Walmajarri people in the area of the Paruku Indigenous Protected Area, which encompasses part of the western range of *Solanum ossicruentum*. Although the authors suggest that the fruits (called “kara” in Walmajarri) are consumed by kangaroos, some Walmajarri people also “eat the outside part… but not the inside part” due to the “saltiness or unpleasantness of the fruit.” While the unripened fruits of *Solanum ossicruentum* are fleshy and “salty” tasting (C. Martine, pers. obs.), the bony nature of mature fruits suggests that the usage described here does not relate to this taxon. Instead, it likely represents one of the other Kimberley forms of *Solanum dioicum* sensu lato.

#### Phenology.

Most flowering specimens have been collected from February-July, with fruiting specimens collected in March-September. Seeds germinated for this study were from diaspores collected at the base of plants bearing flowers and immature fruits at Mirima National Park on 1 May 2014. These were assumed to have developed in the previous growth season.

#### Phylogeny.

Previous phylogenetic work including accessions identified as this form (Martine et al. 2006, Martine et al. 2009) suggested that *Solanum ossicruentum* is a member of the “Dioicum Complex,” a set of several dioecious species largely occupying the Kimberley region. Preliminary work using multiple intronic regions (Martine et al. in prep) infers that *Solanum ossicruentum* is either sister to the rest of that group or represents an independent dioecious lineage. It does not appear to form a clade with the other Australian dioecious species of the “Dioicum Complex” or with the dioecious *Solanum asymmetriphyllum* Specht and *Solanum sejunctum* Brennan, Martine & Symon from Kakadu National Park (Brennan et al. 2006, Martine et al. 2006; Särkinen et al. 2013).

#### Etymology.

The name *Solanum ossicruentum* was chosen based on suggestions from middle school students in Lewisburg, Pennsylvania, USA. In the spring of 2015, CTM presented live plants of the taxon to an assembly of 150 seventh-grade life science students at Donald H. Eichhorn Middle School. The students, with the help of Mr. Bradley Catherman, were invited to examine the plants, ask questions, and then submit an essay proposing and justifying a potential Latin name for the putative new species. Numerous students were drawn to and suggested names based on the characteristics of the fruits, which stain blood red when cut open before maturity and then mature to a dry, bony condition. Thus *ossi*- is used for “bone” and -*cruentum* for “bloody.”

#### Preliminary conservation status.

Based on IUCN Red List Categories (IUCN 2011), *Solanum ossicruentum* is considered Data Deficient (DD). While the species appears to be relatively widespread over a range of approximately 90,000 km^2^, its range is not comprehensively understood. A relatively small number of collections, coupled with the fact that populations often consist of multiple individuals, suggest that the species is common in some localities but uncommon on the regional and global scales. Further data are required before a certain conservation status can be determined. Like other dioecious species of clonal nature, “populations” of *Solanum ossicruentum* have the potential to represent large multi-stemmed genets connected by an underground network of stolons (e.g. Martine et al. 2013). Given that individual genets in dioecious taxa cannot self-fertilize, clonal individuals have particular potential to be reproductively isolated. Recent observations of a small unisexual population by CTM in the Carr Boyd Ranges (just north of Lake Argyle) found that numerous female flowers had bloomed and senesced, ostensibly for lack of nearby male individuals and/or effective pollinators, and preliminary results from a population genetics study (Cantley et al. in prep) show low levels of genetic diversity for the species in Mirima National Park – a surprising outcome given that dioecious taxa are obligate outcrossers.

#### Specimens examined.

**AUSTRALIA. Northern Territory**: Jellebra Rockhole, 19°21'45"S, 129°00'35"E, 7 June 1996, *D.E. Albrecht 7756* (DNA, NT); Cockatoo Creek, Keep River area, 15°55'17"S, 129°03'31"E, 2 September 1974, *Gibbs & Fox 618* (DNA, NE); Spirit Hills, 15°24'58"S, 129°28'39"E, 17 April 2007, *R.A. Kerrigan 1226* (DNA); 11 km east of NE Mt. Frederick, 19°37'S, 129°21'E,1 March 1981, *P.K. Latz 8597* (DNA, NT); Pargee Range, 19°36'S, 129°16'E, 2 April 1981, *P.K. Latz 8608* (DNA, ADW); 8 km SSW Victoria River Bridge, 15°40'47"S, 131°5'34"E, 16 April 1996, *P.K. Latz 14760* (DNA, NT, AREF); Cow Creek, Victoria River, Gregory National Park, 15°52'26.8"S, 131°19'58.6"E,2 May 2001, *C.P. Mangion & G. Boehme 1060* (DNA); Winnecke Hills, 18°37'11"S, 130°16'30"E, 1 May 2004, *C.P. Mangion & D.L. Lewis 1607* (DNA); Nigli Gap Walk, Keep River National Park, 15°45'30.4"S, 129°05'07.4"E, 26 May 2004, *C.T. Martine & W.R. Barker 772* (DNA, CONN); Gurrundalng Walk, Keep River National Park, 15°52'07.8"S, 129°03'11.1"E, 27 May 2004, *C.T. Martine & W.R. Barker 781* (DNA, CONN); 63 km S of Lajamanu, 18.39°S, 130.16°E, 10 Feb 1988, *T.M. Orr 57* (DNA); Mornington Station, 17°33'02"S, 132°01'15"E, 11 April 2004, *JA Risler & S. Legge 2673* (DNA); Bradshaw Military Training Area, 15°04'50"S, 129°33'28"E, 2 April 2007, *B.M. Stuckey & I.D. Cowie 64* (DNA, NSW); 165.8 km NE of Tanami, 18°33'S, 130°10'E, 18 May 1971, *D.E. Symon 6938* (DNA, NT, CANB, PERTH); **Western Australia**: North end of Ragged Range, 16°31'32"S, 128°23'21"E, 17 July 2001, *D.J. Edinger 2601* (DNA, PERTH); 1 mile N of Revolver Creek, Carr Boyd Ranges, 16°14'S, 128°34'E, 13 March 1978, *T.G. Hartley 14561* (DNA, CANB); Sturt Creek Station, 19°18'S, 128°19'E, 20 July 1973, *P.K. Latz* (DNA, NT, ADW, PERTH); Mirima National Park, 15°47'14.1"S, 128°45'37.0"E, 28 May 2004, *C.T. Martine & W.R. Barker* 787 (DNA, CONN); Carr Boyd Ranges, 16°05.207'S, 128°45.406'E, 3 May 2014, *C.T. Martine & R.F. Martine 4057* (DNA, BUPL).

#### Diagnostic couplet.

A comprehensive key to the “Diocum Complex,” including numerous newly recognized species, is forthcoming (Barrett and Barrett in prep). At present, the most complete diagnostic key for the species of the Kimberley region is the key in Barrett (2013), which lumps the primary variations of *Solanum diocium* sensu lato as a single taxon. The following couplet may be inserted where *Solanum dioicum* occurs at couplet 60.

**Table:** 

60a	Plants less than 1 m tall, many-branched; stems moderately prickly; leaf indumentum silvery or rusty, overall aspect silvery-green, yellowish-green, or reddish-green; stigma deeply bifurcating, lobes 2-5 mm; calyx lobes slightly exceeding corolla and enclosed fruits; fruits green and fleshy at maturity	***Solanum dioicum* W.V. Fitzg.**
60a	Plants 1–2 m tall, few-branched and conspicuously Y-shaped in form; stems exceptionally prickly; leaf indumentum silvery, overall aspect silvery-blue; stigma shallowly bifurcating, lobes 0.5-1 mm; calyx lobes far exceeding corolla and enclosed fruits; fruits bony and dry at maturity	***Solanum ossicruentum* Martine & J. Cantley**

#### Discussion.

*Solanum ossicruentum* has been noted for nearly 50 years as a widespread morphotype of *Solanum dioicum* known as ‘Tanami’ (Symon 1981, Purdie et al. 1982). The outstanding characters noted here, particularly its silvery tomentum, conspicuously long calyx lobes, upright and Y-shaped to inverted tripod-shaped stature, and dioecious breeding system, make it easily recognizable in the field, and its putative trample-burr dispersal syndrome is unusual among allied species. Symon (1979) described the fruits of *Solanum dioicum* sensu lato as belonging to a large group of species with firm, yellowish berries – but he identified a set of six solanums in northern Australia as bearing “trample burr” fruits that are shed when ripe. Notably, Symon included *Solanum leopoldensis* Symon, another member of the “Dioicum Complex,” in this group. The fruits of *Solanum leopoldensis*, like those of *Solanum ossicruentum*, mature to a bony condition and remain enclosed in a spiny calyx. The recently described *Solanum zoeae* R.L. Barrett is closely allied with *Solanum leopoldensis* and shares similar fruiting characteristics (Barrett 2013); and the forthcoming recognition of a number of new dioecious *Solanum* species in the Kimberley (Barrett and Barrett in prep) may provide evidence that “trample burr” morphology is more widespread than currently thought.

In overall aspect, the new species most closely resembles *Solanum beaugleholei* Symon and *Solanum phlomoides* A. Cunn. ex Benth. (both endemic to NW Australia) based on leaf morphology, tomentum, and coloration, but both of these species are less rigidly upright, have much larger (only partially enclosed) fleshy fruits, and exhibit an andromonoecious breeding system.

Recent surveys in remote regions of the Kimberley suggest that the total number of dioecious taxa in that region may be around 20 (Barrett 2013, M. Barrett pers. comm.), with three other named dioecious species endemic to the Northern Territory: *Solanum asymmetriphyllum*, *Solanum cowiei* Martine (Martine et al. 2014), and *Solanum sejunctum* (Brennan et al. 2006). The prevalence of functional dioecy among the solanums of Australia, relative to the few other incidences recorded elsewhere (Knapp 1998, Martine and Anderson 2007), continues to be of great interest and will be further informed by ongoing work in reproductive ecology (e.g., Martine and Anderson 2008; Jordon-Thaden et al. in prep), population genetics (Cantley et al. in prep.), and phylogenomics (Martine et al. in prep). It is hoped that these and other studies (e.g., Barrett and Barrett in prep) will help resolve the problematic taxonomy of *Solanum dioicum* sensu lato, a nomenclatural issue that currently impedes efforts to recognize and protect the true biodiversity of *Solanum* in northwestern Australia.

## Supplementary Material

XML Treatment for Solanum ossicruentum
